# An outbreak of hantavirus pulmonary syndrome, Chile, 1997.

**DOI:** 10.3201/eid0404.980425

**Published:** 1998

**Authors:** J Toro, J D Vega, A S Khan, J N Mills, P Padula, W Terry, Z Yadón, R Valderrama, B A Ellis, C Pavletic, R Cerda, S Zaki, W J Shieh, R Meyer, M Tapia, C Mansilla, M Baro, J A Vergara, M Concha, G Calderon, D Enria, C J Peters, T G Ksiazek

**Affiliations:** Ministry of Health, Santiago, Chile.

## Abstract

An outbreak of 25 cases of Andes virus-associated hantavirus pulmonary syndrome (HPS) was recognized in southern Chile from July 1997 through January 1998. In addition to the HPS patients, three persons with mild hantaviral disease and one person with asymptomatic acute infection were identified. Epidemiologic studies suggested person-to-person transmission in two of three family clusters. Ecologic studies showed very high densities of several species of sigmodontine rodents in the area.


### An Outbreak of Hantavirus Pulmonary Syndrome, Chile, 1997

**Jorge Toro,* Jeanette D. Vega,† ‡‡ Ali S. Khan,‡ James N. Mills,‡ Paula Padula,§ William Terry,‡ Zaida Yadón,§ Rosa Valderrama,¶ Barbara A. Ellis,‡ Carlos Pavletic,* Rodrigo Cerda,† Sherif Zaki,‡ Shieh Wun-Ju,‡ Richard Meyer,‡ Mauricio Tapia,# Carlos Mansilla,# Michel Baro,** Jose A. Vergara,** Marisol Concha,* Gladys Calderon,†† Delia Enria,†† C.J. Peters,‡ and Thomas G. Ksiazek‡**

*Ministry of Health, Santiago, Chile; †Pan American Health Organization, Santiago, Chile; ‡Centers for Disease Control and Prevention, Atlanta, Georgia, USA; §Instituto Nacional de Enfermedades Infecciosas ANLIS "Carlos G. Malbran," Buenos Aires, Argentina; ¶Aysen Region XI Health Service, Coyhaique, Chile; #Coyhaique Regional Hospital, Coyhaique, Aysen, Chile; **Llanchipal Health Services, Puerto Montt, Chile; ††Instituto Nacional de Enfermedades Virales Humanas, Pergamino, Argentina; and ‡‡Catholic University of Chile, Santiago, Chile.

An outbreak of 25 cases of Andes virus-associated hantavirus pulmonary syndrome (HPS) was recognized in southern Chile from July 1997 through January 1998. In addition to the HPS patients, three persons with mild hantaviral disease and one person with asymptomatic acute infection were identified. Epidemiologic studies suggested person-to-person transmission in two of three family clusters. Ecologic studies showed very high densities of several species of sigmodontine rodents in the area.

Hantavirus pulmonary syndrome (HPS), first recognized in 1993 after a cluster of acute respiratory distress syndrome deaths in the southwestern United States, is now considered a pan-American zoonosis (1,2). HPS is characterized by fever, myalgia, gastrointestinal symptoms, and headache, with subsequent characteristic cardiopulmonary dysfunction and a 40% to 60% case-fatality rate (3). Although the etiologic agent, Sin Nombre virus (SNV), causes clinical symptoms that appear different from those that Eurasian hantaviruses cause in hemorrhagic fever with renal syndrome, SNV shares many features with these Old World hantaviruses (4,5), including an association with a single primary rodent host *(Peromyscus maniculatus* [deer mouse] in the case of SNV), which acts as the natural reservoir (6).

After SNV was identified, numerous other New World hantaviruses were rapidly identified throughout the Americas by reverse transcription-polymerase chain reaction (RT-PCR) amplification of viral RNA from captured antibody-positive rodents and, occasionally, from infected patients (2). HPS was first identified in Chile in 1995 in the Cochamo, Los Lagos region. Genetic sequencing of RT-PCR products from the autopsy tissues of another patient presumptively infected in this region in 1996 confirmed infection with Andes virus (7), which had previously been identified in southern Argentina as a cause of HPS, with *Oligoryzomys longicaudatus* postulated as the reservoir host (8). Sporadic cases were reported in Chile until an outbreak that included two family clusters was recognized in the Coyhaique Health District, Aysen region, in August 1997; a third cluster was reported in January 1998. This outbreak prompted a joint investigation by the National Administration of Laboratories and Institutes of Health (Argentina), Pan American Health Organization, Centers for Disease Control and Prevention (CDC, USA), in collaboration with the Ministry of Health of Chile, to define the epidemiology and ecology of the syndrome in Chile.

Address for correspondence: Ali S. Khan, CDC, Mail Stop A26, 1600 Clifton Road, Atlanta, GA, 30333, USA; fax: 404-639-1509, e-mail: ask0@cdc.gov.

### The Investigation

### Epidemiologic Surveillance and Case Identification

National and local surveillance for HPS was reinforced with explicit case definitions for HPS, asymptomatic hantavirus infection, and mild hantaviral disease. HPS was defined as an acute febrile illness (temperature >38.3°C) characterized by unexplained acute respiratory distress syndrome or bilateral interstitial pulmonary infiltrates, with respiratory compromise requiring supplemental oxygen, or an unexplained illness resulting in death, with autopsy results showing noncardiogenic pulmonary edema without an identifiable specific cause of death. In addition to a compatible clinical illness, the following laboratory evidence of infection was required: 1) hantavirus-specific immunoglobulin M (IgM) or a fourfold rise in IgG titer, or 2) positive RT-PCR results for hantavirus RNA, or 3) positive immunohistochemical results for hantavirus antigen.

Asymptomatic hantavirus infection was defined as laboratory evidence of acute hantavirus infection (presence of IgM antibodies) in persons with no documented concurrent illness. Mild hantaviral disease was defined as IgM-positive results and a febrile illness without objective pulmonary dysfunction (i.e., hypoxemia and radiographic abnormalities). All other persons with isolated detectable hantavirus-specific IgG antibodies were classified as “other seropositives.”

All available clinical charts and case reports from the Aysen region were reviewed in conjunction with interviews of family members from the two described clusters and survivors. A third cluster was identified in January 1998 and was also reviewed.

### Ecologic Studies

Rodents were trapped at two principal sites near the residence sites of the first two reported family clusters, as well as in several areas of less disturbed native vegetation in Lago Atravesado. Traps were placed at each of the two case-households, as well as at two neighboring controls for each case-household (one within 500 m and the other 500 m to 1,000 m). Each evening, 150 to 200 live-capture traps were placed within and around residences, outbuildings, gardens, and woodpiles, along fence lines, and within remnant patches of native vegetation. Traps were checked in the early morning, and samples of blood and organ tissues were collected according to standardized protocols (9). For comparison, we used similar trapping techniques at additional sites near Valdivia (700 km north of the outbreak area) and the capital city of Santiago (1,400 km north). Rodent taxonomy is as described by Musser and Carleton, 1993 (10).

### Laboratory Testing

Human serum specimens were tested for IgG antibodies reactive with SNV and Andes antigens by an enzyme-linked immunosorbent assay (ELISA) (11). Rodent whole-blood specimens were similarly tested for IgG antibodies to SNV. An IgM-capture ELISA was performed on human sera by using inactivated Laguna Negra virus at CDC and an IgM-capture ELISA with recombinant Andes nucleocapsid antigen at the National Institute for Infectious Disease, Buenos Aires, Argentina (P. Padula, unpub. data). Immunohistochemical analysis for hantaviral antigens was performed by using a cross-reactive monoclonal antibody directed against conserved hantaviral nucleocapsid epitopes (12). Specimens from HPS patients were also examined for viral genetic materials (M and S segment) by nested and heminested RT-PCR from either autopsied tissues or blood clot samples (13,14). Genetic sequencing of RT-PCR products derived from patient samples was also performed.

### The Outbreak

From October 1995 when HPS was first recognized in Chile through June 1997, seven additional cases were identified (Figure 1). Between July 1, 1997, and January 22, 1998, 25

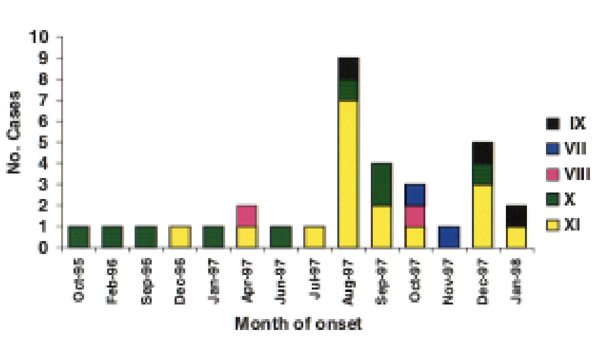

Figure 1. Temporal distribution of hantavirus pulmonary syndrome cases, Chile, 1995-1998.

HPS patients and three family clusters were identified in Chile. By RT-PCR we confirmed that 16 of these patients had been infected with Andes virus. Except for six cases, the recent outbreak was centered in Regions X (Llanchipal) and XI (Aysen) (combined population = 1.1 million) (Figure 2). The areas where these patients lived are sparsely populated, with numerous lakes, in a *Nothofagus* (southern beech) forest ecosystem. From October 4, 1995, to January 22, 1998, a total of 33 cases were identified in Chile, with a case-fatality rate of 54%. The mean age was 31.4 years (range: 1 year 11 months to 60 years); five (15.2%) of the patients were children under 17 years of age; and 76% of the patients were male. No cases were reported among health-care workers.

No significant difference in mean age, case-fatality rate, or proportion of male patients was evident between the July to January outbreak and previous cases; however, all the pediatric cases occurred during the July to January outbreak.

### Family Clusters

The first family cluster (Cluster 1) of HPS cases was reported from Cisne Medio, Lago Verde community, Aysen region. The 39-year-old male head of the family became ill with an acute febrile illness on July 15 and died in transit to the regional hospital on July 21; HPS was subsequently confirmed by immunohistochemistry and RT-PCR (M and S segment), with a genetic sequence consistent with Andes virus. The family moved to the village of Villa Amengual, 7 km away, on July 22 and remained there except for a brief visit to Cisne Medio on July 27 to retrieve personal belongings. The wife of the index patient became ill with HPS on August 2 and died on August 8. Her 2-year-old and 12-year-old sons became ill 1 week and 16 days, respectively, after she became ill. HPS was confirmed in both children by serologic testing; both children survived. The brother-in-law of the index patient, who continued to reside intermittently in the original household, became ill 33 days after his sister became ill and died (Figure 3). Thus, the immediate family members became ill 12, 19, and 28 days after leaving the family homestead, and the intervals between the onset of the index and later cases were 18, 25, and 34 days.

In contrast, the second family cluster (Cluster 2) included all four members of a household in Lago Atravesado, Coyhaique community, Aysen region, who became ill within 5 days of each other; one of them, a 2-year-old child with no respiratory symptoms, was classified as having a mild hantaviral infection. The third family cluster (Cluster 3) included a husband, who worked in a rural area, and his wife, who remained in the family home in urban Coyhaique, a city of 60,000 inhabitants. The husband became ill with symptoms suggestive of HPS 12 days after returning to his family home. He was hospitalized and died on December 19.

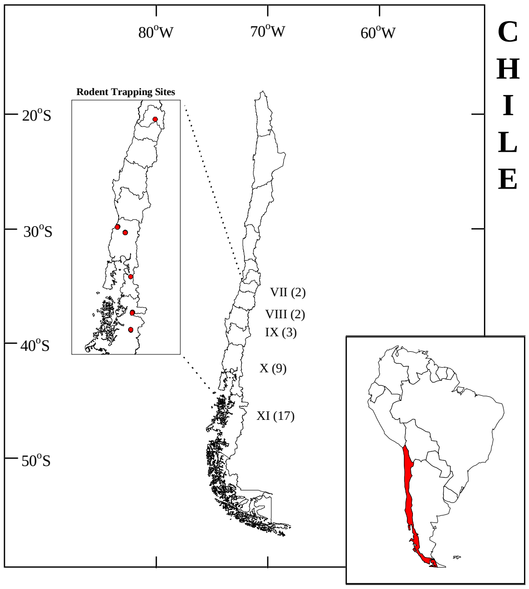

Roman numerals indicate designated regions; numbers in parenthesis give corresponding numbers of confirmed HPS cases in each region.

Figure 2. Geographic distribution of hantavirus pulmonary syndrome cases, Chile, 1995-1998.

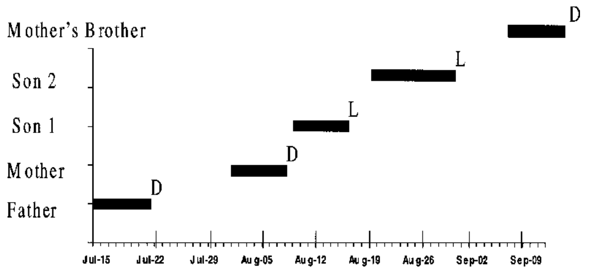

Figure 3. Time-line listing for hantavirus pulmonary syndrome cases in Cluster 1, Chile, 1997.

His wife became ill 22 days after the onset of the husband's symptoms. She had not traveled outside the town of Coyhaique during the previous 12 months and reported no exposure to rodents or their excreta. The only known exposures for the wife were washing her husband's clothing and caring for him while he was ill. She survived and was discharged from the hospital on January 28.

Genetic sequencing of viral RNA from cases of immediate family members from Clusters 1 and 2 demonstrated 3.6% divergence in a 167-nucleotide G2 fragment between the clusters. However, the genetic sequence was identical within each of the two clusters. Genetic sequencing of viral RNA from an additional patient from Cisne Medio not related to the family members in Cluster 1, and the brother-in-law in Cluster 1, demonstrated a distinct G2 sequence, with 1%-4% sequence divergence between them (P. Padula, unpub. data).

### Clinical and Histopathologic Features

In general, the clinical description of HPS cases in Aysen was similar to that of the cases in North America. However, included in the Aysen cases were three children with petechiae; one of them died rapidly with hemorrhagic pulmonary secretions and bleeding from puncture sites. Six of the seven patients on whom urinanalysis was performed had microscopic hematuria and casts; three had proteinuria of >100 mg/dl. In addition, one child in the Llanchipal region had concurrent acute rubella infection. One of the most recent cases occurred in Maule, Region VII, in a 23-week pregnant woman who had typical HPS symptoms and fetal death. Subsequently, disseminated intravascular coagulopathy developed and the woman died of multisystem failure.

The histopathologic features in the lung of HPS case-patients included an interstitial mononuclear infiltrate and intraalveolar edema. Immunohistochemical staining showed hantaviral antigens in the microvascular endothelial cells of the lung and other tissues—typical SNV-associated HPS cases. Prominent immunostaining of pulmonary macrophages was also noted in some cases. A unique feature in the series was fine granular immunostaining in the hepatic cells in some cases, a pattern not observed in a large series of North American HPS cases previously examined (Figure 4) (12).

### Laboratory Test Results

Serologic testing was conducted for all patients and contacts of patients with confirmed HPS cases in Aysen. Among 53 contacts of 14 patients, two (3.8%) had serologic evidence of an acute infection (one had no illness, and another [described previously in cluster 2] had a mild febrile illness without pulmonary disease that did not meet the HPS case definition), and one was IgG positive. In addition, two hospitalized patients had mild hantaviral disease (M. Tapia, unpub. data). Test results of specimens (SNV and Andes antigens were used) were 100% concordant. Nested and heminested RT-PCR assays were conducted for 20 (71.4%) of 28 HPS cases; all 20 were positive. Sixteen (57.1%) were sequenced and characterized as Andes virus.

### Ecologic Studies

A total of 253 rodents were captured during 3 nights of trapping (574 trap nights) in the vicinity of Coyhaique. Overall trap success (captures per 100 trap nights) at case and control home sites was 37% at Cisne Medio and 50% at Lago Atravesado. Trap success at Cisne Medio within and adjacent to the case-household was 66%, compared with only 18% at control homes; this difference may reflect the use of rodenticides in and around the control households. Trap success at distances greater than 100 m from the households was more similar between case and controls (63% versus 43%). The most frequently captured rodent species was *O. longicaudatus*, which comprised 47% of the captured rodents in the area; 13 (12.7%) of 102 tested were hantavirus-antibody–positive. *Akodon olivaceus* comprised a further 33% of the captures (6 [7.5%] of 80 were antibody-positive), and 16% were *Akodon longipilis* (1 [2.7%] of 36 antibody-positive). Eight of 10 rodents captured inside the case-home at Cisne Medio were *O. longicaudatus,* and one was hantavirus-antibody–positive. High trap success in a forested area near Lago Atravesado (22 captures in 40 trap nights, or 55%) indicated that high rodent population densities were not restricted to peridomestic areas.

Trap success was moderate near Valdivia (52 captures from 660 trap nights, or 8% trap success) and very low near Santiago (8 captures from 453 trap nights, or <2%). The species composition was also very different from that encountered in the Coyhaique area: despite

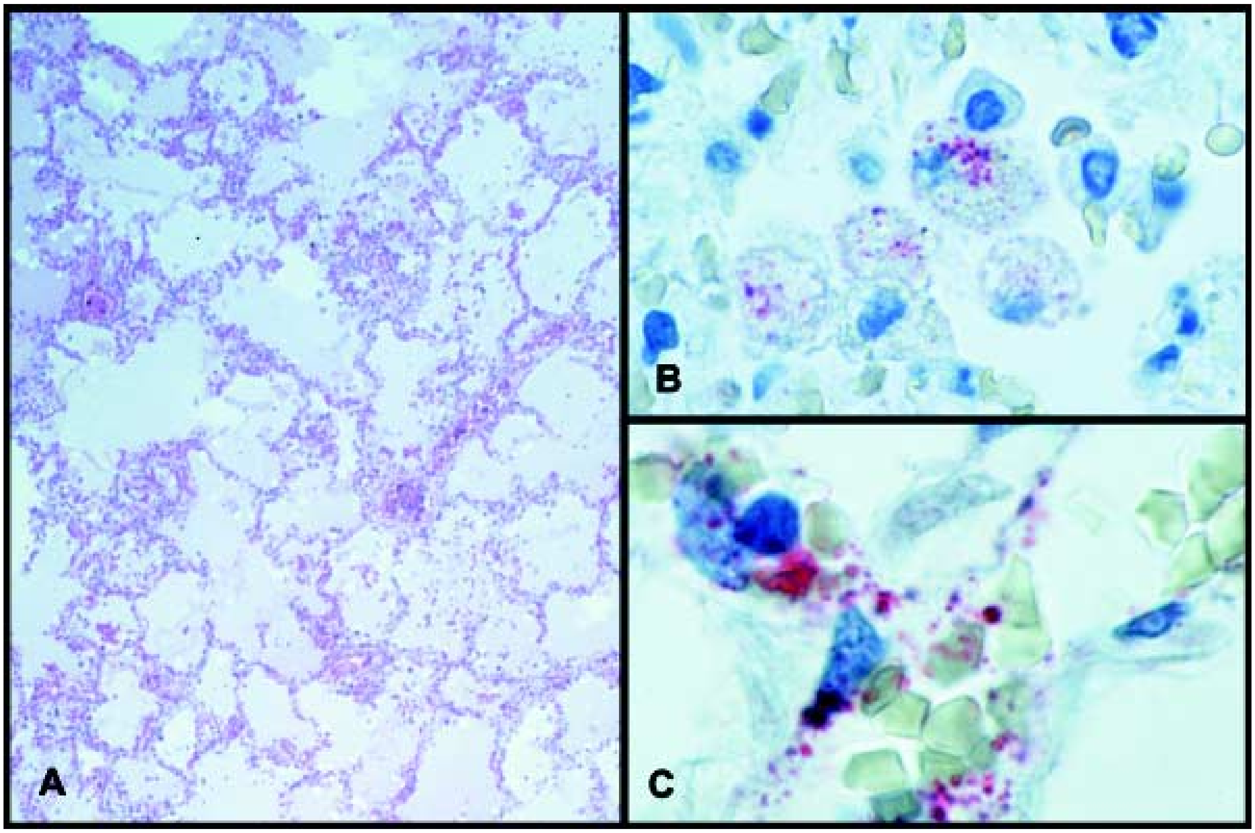

Figure 4. A) Low-power photomicrograph of lung showing interstitial pneumonitis and intraalveolar edema. B) Andes virus antigen–positive intraalveolar macrophages. C) Fine granular immunostaining of hantaviral antigens in endothelial cells of pulmonary microvasculature. (A, Hematoxylin and eosin; B,C, napthol-fast red with hematoxylin counterstain; original magnifications A x 50, B and C x 250.)

trapping in rural areas, 31% of rodents captured near Valdivia and Santiago were the murine species, *Rattus rattus, R. norvegicus,* and *Mus musculus*. No hantavirus-antibody–positive rodents were found from Valdivia or Santiago.

### Conclusions

This outbreak of HPS and the presence of endemic disease in Chile support the assumption that HPS will continue to occur throughout the Americas (range of the sigmodontine rodents that host the genetic group of HPS-causing hantaviruses). In Latin America, endemic HPS and epidemics have been reported in Argentina, Brazil, and Paraguay (15-19). Sporadic cases and SNV-like viral sequences have been reported in these countries and in Bolivia and Uruguay (P. Padula, pers. comm.,7,20). Retrospective studies have verified that HPS cases occurred in the United States as early as 1959 (21). The factors responsible for the outbreaks in the Americas have not been well defined and may not be the same in all regions. However, periodic climatic and ecologic events cause dramatic local increases in rodent reservoir populations, known as "irruptions" or "ratadas" (22) and may lead to an increase in the number of rodent-human contacts.

Small-mammal trap success in the Coyhaique area was 5 to 30 times higher than in rural areas 700 km to 1,400 km further north. These extremely high trap success values indicate a rodent irruption in southern Chile whose causes may be related to the flowering of a species of bamboo and a relatively benign preceding winter (22). Regardless of causes, however, the extremely high rodent densities and evidence of hantavirus infection in the two most common species strongly implicate contact with rodents as the primary mode of disease transmission to humans. The rodent species with the highest antibody prevalence, *O. longicaudatus*, is the reservoir for Andes virus in Argentina and Chile (8,23). No hantavirus has been associated with *A. olivaceus*. RT-PCR conducted on tissue samples from seropositive animals yielded amplifiable viral RNA from seven of seven *O. longicaudatus*, two of four *A. olivaceus*, and one of one *A. longipilis*. Sequencing showed that all PCR products represented Andes virus, and sequences were nearly identical among all three species (S. Morzunov, unpub. data). The near identity between sequences from all three species and the fact that two of four samples from *A. olivaceus* were PCR negative suggest that hantavirus infection in *A. olivaceus* and *A. longipilis* represents spillover of virus from the primary reservoir, *O. longicaudatus*. The absence of viral RNA in two of the four samples from *A. olivaceus* also suggests that the infection is of shorter duration in this presumably nonhost species.

A number of features may eventually differentiate HPS caused by Andes virus from that caused by other New World viruses. Several mild and asymptomatic hantavirus infections have been reported in Argentina and Chile, whereas in North America the case-to-infection ratio is approximately 1:1 (24,25). In this investigation, four (11%) of the patients with serologic evidence of acute infection did not have HPS. More mild and asymptomatic disease in some South American hantavirus infections, in addition to infection with nonpathogenic hantaviruses, may be among the factors explaining antibody prevalence as high as 40% among indigenous persons of Argentina (C. Johnson, unpub. data). Similar case-to-infection ratios may explain the high prevalence (35% to 57%) of hantavirus antibodies in some regions of Paraguay (16,26).

Disease in Chile has also been characterized by an increased propensity toward bleeding, with petechiae in 50% of the pediatric cases and increased renal involvement with microscopic hematuria and cellular casts in the three patients whose urinanalysis results were available. Flushing of the head and upper thorax is indicative of vascular dysregulation and is a well-described sign in hemorrhagic fever with renal syndrome. It has also been described in Andes virus-infected patients from the adjacent areas of Argentina (17,27), but not in Chile.

The relatively large proportion of children with HPS reported in Chile is unexplained. Infection in children is relatively rare in the United States, where only 8 (4.5%) of 179 reported cases were in children 16 years old or younger. Moreover, this dearth of pediatric cases has also been reported for hemorrhagic fever with renal syndrome (28). It is unclear if this greater incidence of pediatric cases is Andes virus- or outbreak-specific. However, Argentina has also seen a larger proportion of pediatric HPS cases than the United States, caused by three different genotypes: Andes, Oran, and Lechiguanas.

Immunostaining showed hantaviral antigens in the microvascular endothelial cells of the lung and other tissues with prominent staining of pulmonary macrophages in some cases. A unique feature not previously described in SNV-associated HPS cases was fine, granular immunostaining in the hepatic cells of three patients. The specificity of this hepatic staining is being investigated. In Argentina, person-to-person transmission has been demonstrated in at least one epidemic of Andes virus infection (17). Although the mechanism of that transmission is unknown, the increased staining of intraalveolar pulmonary macrophages suggests the presence of virus within the alveolar space with the potential for small-particle aerosolization. A comparison of disease variants that may be hantavirus-strain–specific will be required to address this hypothesis.

No discrepancies were observed in serologic results from testing with SNV or Andes antigens, which underlines the current recommendation that diagnosis be based on serologic testing, especially given the difficulties in handling and transporting fresh tissues for RT-PCR and the possibility of cross-contamination. Formalin-fixed tissues from case-patients who died should be used for immunohistochemical tests, particularly if no serum or blood was obtained. However, even in fatal cases, an attempt should be made to obtain heart blood for serologic tests.

Person-to-person transmission cannot be excluded in the first or third family cluster. We did not trap rodents within the town limits at Villa Amengual or in the city of Coyhaique. However, although large numbers of rodents were seen in rural areas, rodent infestation in Villa Amengual or Coyhaique had not been reported. Further, since *O. longicaudatus* is a sylvatic species not generally associated with urban habitats, subsequent exposure to infected

*O. longicaudatus* was likely not responsible for the wife's illness in family cluster 3. However, as in Cluster 2, Andes and other associated hantaviruses can cause clustering attributable to a single point–source contact with infected rodents.

Person-to-person transmission of Andes virus-associated HPS has been documented (17,29), although this type of transmission has not been observed with SNV-associated HPS (24,30). The lack of cases among health-care workers suggests that current protective methods are adequate; however, standard precautions should be reinforced. Until the propensity of Andes virus to be transmitted from person to person is clarified, patients with suspect cases should be isolated in separate rooms, contact and droplet precautions should be used, and respiratory precautions should be considered.

### Acknowledgments

We thank Drs. Alex Figueroa, Roberto Belmar, Hugo Salinas, and J. Montecinos for their support to complete these studies, and John O'Connor for editorial review of this manuscript. Dr. Roberto Murua provided valuable support of rodent trapping efforts, and Dr. Milton Gallardo confirmed the rodent species identifications.

Dr. Toro is an epidemiologist with the Chilean Ministry of Health, professor of public health at the Universidad de Chile in Santiago, and director of the Chilean Society of Epidemiology.

### References

1. Duchin JS, Koster FT, Peters CJ, Simpson GL, Tempest B, Zaki SR, et al. Hantavirus pulmonary syndrome: a clinical description of 17 persons with a newly recognized disease. N Engl J Med 1994;330:949-55.
2. Khan AS, Ksiazek TG, Peters CJ. Hantavirus pulmonary syndrome. Lancet 1996;347:739-41.
3. Khan AS, Khabbaz RF, Armstrong LR, Holman RC, Bauer SP, Graber J, et al. Hantavirus pulmonary syndrome: the first 100 U.S. cases. J Infect Dis 1996;173:1297-303.
4. Ksiazek TG, Peters CJ, Rollin PE, Zaki S, Nichol S, Spiropoulou C, et al. Identification of a new North American hantavirus that causes acute pulmonary insufficiency. Am J Trop Med Hyg 1995;52:117-23.
5. Nichol S, Spiropoulou C, Morzunov S, Rollin P, Ksiazek T, Feldmann H, et al. Genetic identification of a hantavirus associated with an outbreak of acute respiratory illness. Science 1993;262:914-7.
6. Childs JE, Ksiazek TG, Spiropoulou CF, Krebs JW, Morzunov S, Maupin GO, et al. Serologic and genetic characterization of *Peromyscus maniculatus* as the primary rodent reservoir for a new hantavirus in the southwestern United States. J Infect Dis 1994;169:1271-80.
7. Espinoza R, Vial P, Noriega LM, Johnson A, Nichol ST, Rollin PE, et al. Hantavirus pulmonary syndrome in a Chilean patient with recent travel in Bolivia. Emerg Infect Dis 1998;4:93-4.
8. Levis SC, Morzunov SP, Rowe JE, Enria D, Pini N, Calderon G, et al. Genetic diversity and epidemiology of hantaviruses in Argentina. J Infect Dis 1998;177:529-38.
9. Mills JN, Childs JE, Ksiazek TG, Peters CJ, Velleca WM. Methods for trapping and sampling small mammals for virologic testing. Atlanta (GA): U.S. Department of Health and Human Services; 1995. p. 61.
10. Musser GG, Carleton MD. Family Muridae. In: Wilson DE, Reeder DM, editors. Mammal species of the world, a taxonomic and geographic reference. Washington: Smithsonian Institute; 1993. p. 501-755.
11. Feldmann H, Sanchez A, Morzunov S, Spiropoulou CF, Rollin PE, Ksiazek TG, et al. Utilization of autopsy RNA for the synthesis of the nucleocapsid antigen of a newly recognized virus associated with hantavirus pulmonary syndrome. Virus Res 1993;30:351-67.
12. Zaki SR, Greer PW, Coffield LM, Goldsmith CS, Nolte KB, Foucar K, et al. Hantavirus pulmonary syndrome: pathogenesis of an emerging infectious disease. Am J Pathol 1995;146:552-79.
13. Lopez N, Padula P, Rossi C, Lazaro ME, Franze-Fernandez MT. Genetic identification of a new hantavirus causing severe pulmonary syndrome in Argentina. Virology 1996;220:223-6.
14. Hjelle B, Jenison S, Torrez-Martinez N, Yamada T, Nolte K, Zumwalt R, et al. A novel hantavirus associated with an outbreak of fatal respiratory disease in the southwestern United States: evolutionary relationships to known hantaviruses. J Virol 1994;68:592-6.
15. Da Silva MV, Vasconcelos MJ, Hidalgo NTR, Veiga APR, Canzian M, Marotto PCF, et al. Hantavirus pulmonary syndrome: report of the first three cases in São Paulo, Brazil. Rev Inst Med Trop Sao Paulo 1997;39:231-4.
16. Joachini L, Thompson M, Rojas de Arias A, Iwasaki E, Torrez-Martinez N, Von Bredow C, et al. Prevalence of hantavirus antibodies in select populations of Eastern and Western Paraguay. In: Abstracts of the Fourth International Conference on HFRS and Hantaviruses; 1998 Mar 5-7; Atlanta, Georgia.
17. Wells RM, Sosa ES, Yadon ZE, Enria D, Padula P, Pini N, et al. An unusual hantavirus outbreak in southern Argentina: person-to-person transmission? Emerg Infect Dis 1997;3:171-4.
18. Levis SC, Briggiler AM, Cacase M, Peters CJ, Ksiazek TG, Cortes J, et al. Emergence of hantavirus pulmonary syndrome in Argentina [abstract]. Am J Trop Med Hyg 1995;54 Suppl:441.
19. Williams RJ, Bryan RT, Mills JN, Palma RE, Vera I, Velasquez F, et al. An outbreak of hantavirus pulmonary syndrome in Western Paraguay. Am J Trop Med Hyg 1997;57:274-82.
20. Hjelle B, Torrez-Martinez N, Koster FT. Hantavirus pulmonary syndrome-related virus from Bolivia [letter]. Lancet 1996;347:57.
21. Frampton JW, Lanser S, Nichols CR, Ettestad PJ. Sin Nombre virus infection in 1959 [letter]. Lancet 1995;346:781-2.

22. Murua R, Gonzales LE, Gonzales M, Jofre YC. Efectos del florecimiento del arbusto *Chusquea quila* Kunth (Poaceae) sobre la demografía de poblaciones de roedores de los bosques templados frios del sur Chileno. Boletín de la Sociedad de Biología, Concepción, Chile 1996;67:37-42.
23. López N, Padula P, Rossi C, Miguel S, Edelstein A, Ramirez E, et al. Genetic characterization and phylogeny of Andes virus and variants from Argentina and Chile. Virus Res 1997;50:77-84.
24. Wells RM, Young J, Williams RJ, Armstrong LR, Busico K, Khan AS, et al. Hantavirus transmission in the United States. Emerg Infect Dis 1997;3:361-5.
25. Levis S, Rowe JE, Morzunov S, Enria DA, St Jeor S. New hantaviruses causing hantavirus pulmonary syndrome in Central Argentina [letter]. Lancet 1997;349:998-9.
26. Ferrer JF, Jonsson CB, Esteban E, Calligan D, Basombrio MA, Peralta-Ramos M, et al. High prevalence of hantavirus antibodies in Indian communities of the Paraguayan and Argentinean Gran Chaco. Am J Trop Med Hyg 1998;59:438-44.
27. Parisi MDN, Enria DA, Pini NC, Sabattini MS. Retrospective detection of clinical infections caused by hantavirus in Argentina. Medicina (B Aires)1996;56:1-13.
28. Khan AS, Ksiazek TG, Peters CJ. Viral hemorrhagic fevers among children. Seminars in Pediatric Infectious Diseases 1997;8:64-73.
29. Padula PJ, Edelstein A, Miguel SDL, López NM, Rossi CM, Rabinovich RD. Hantavirus pulmonary syndrome (HPS) outbreak in Argentina: molecular evidences for person-to-person transmission of Andes virus. Virology 1998;241:323-30.
30. Vitek CR, Breiman RF, Ksiazek TG, Rollin PE, McLaughlin JC, Umland ET, et al. Evidence against person-to-person transmission of hantavirus to health care workers. Clin Infect Dis 1996;22:824-6.
